# Supplementary figures and images for: A 3-DoF robotic platform for the rehabilitation and assessment of reaction time and balance skills of MS patients
Source: PLoS One. 2023 Feb 24;18(2):e0280505. doi: 10.1371/journal.pone.0280505 (PMC9955682; doi:10.1371/journal.pone.0280505)

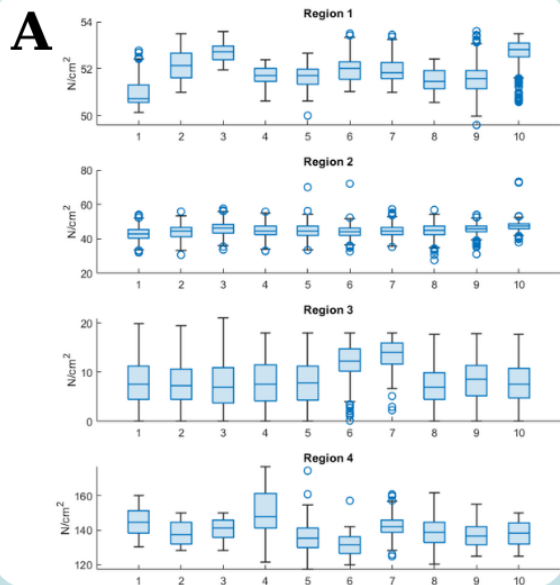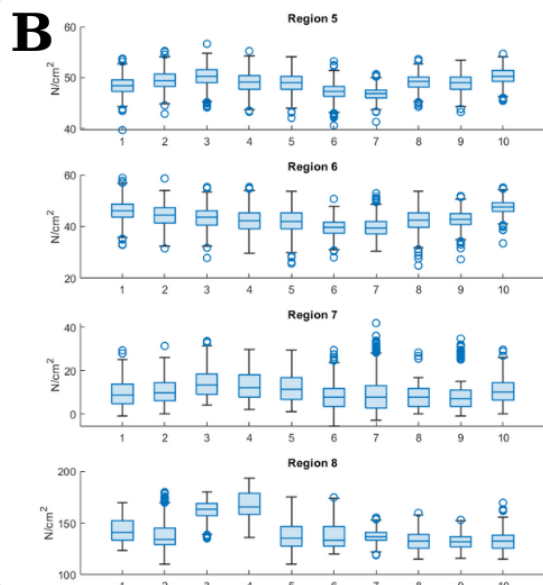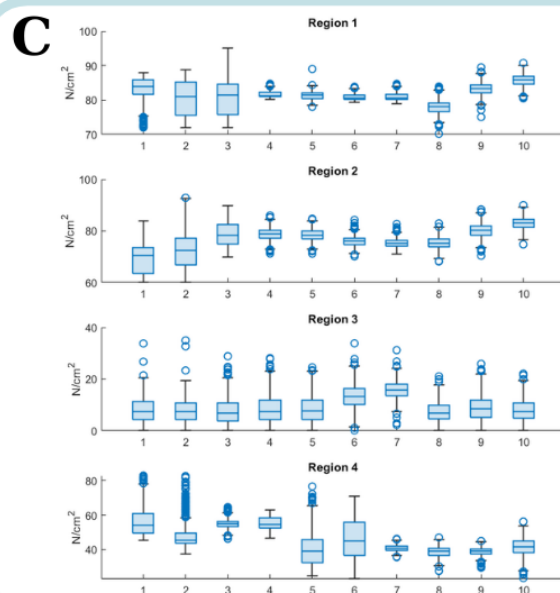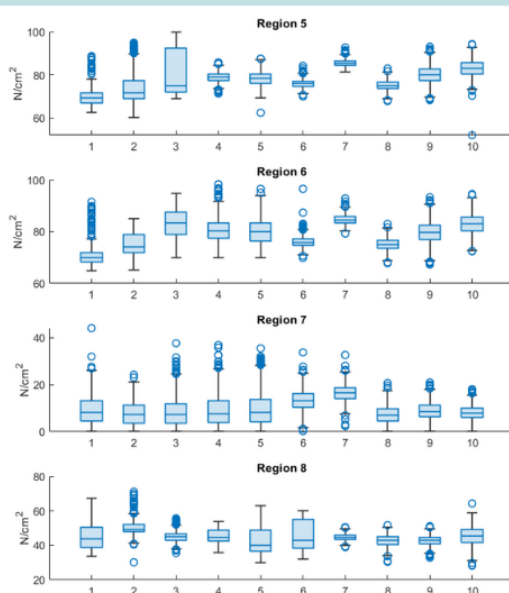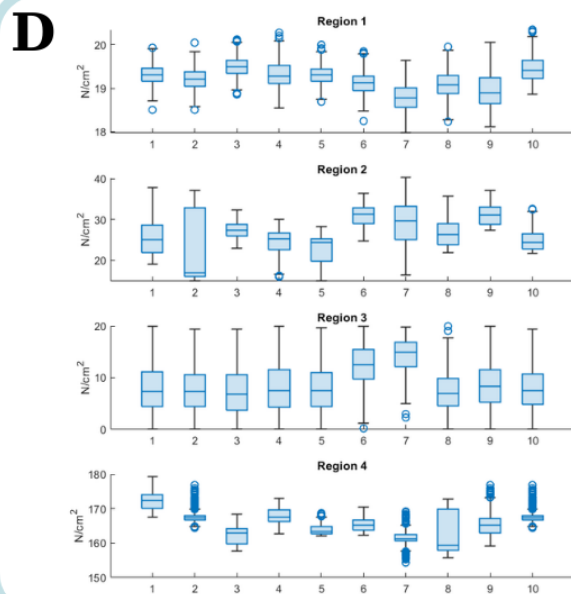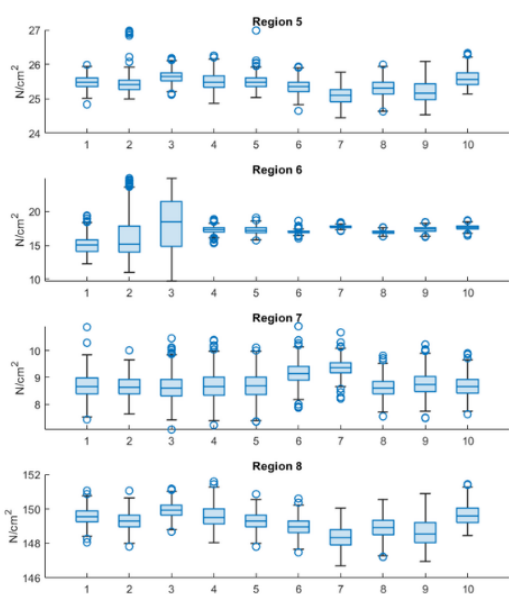

Supplement: S1 Fig — Static Design Evaluation Tests: Pressure distribution of the load cell regions under (A) Case1a: standing on the left foot scenario, (B) Case1b: standing on the right foot scenario, (C) Case2: standing in forefoot scenario, and (D) Case3: standing on the rearfoot scenario. (PDF) [file pone.0280505.s001.pdf]

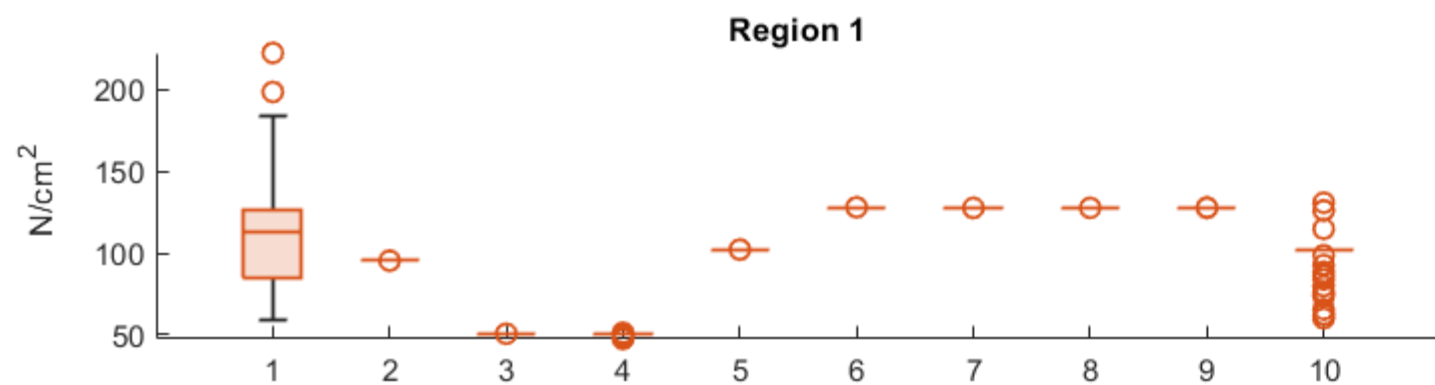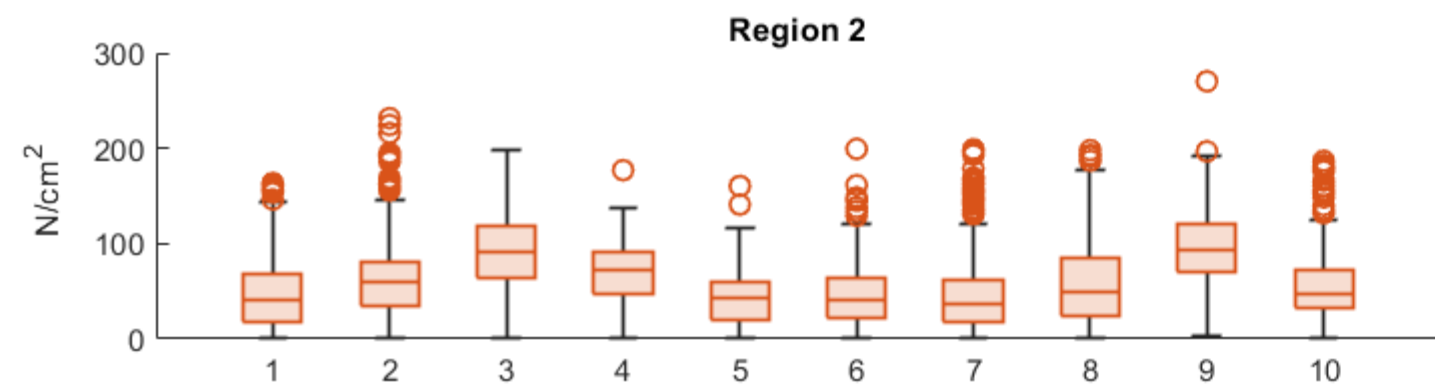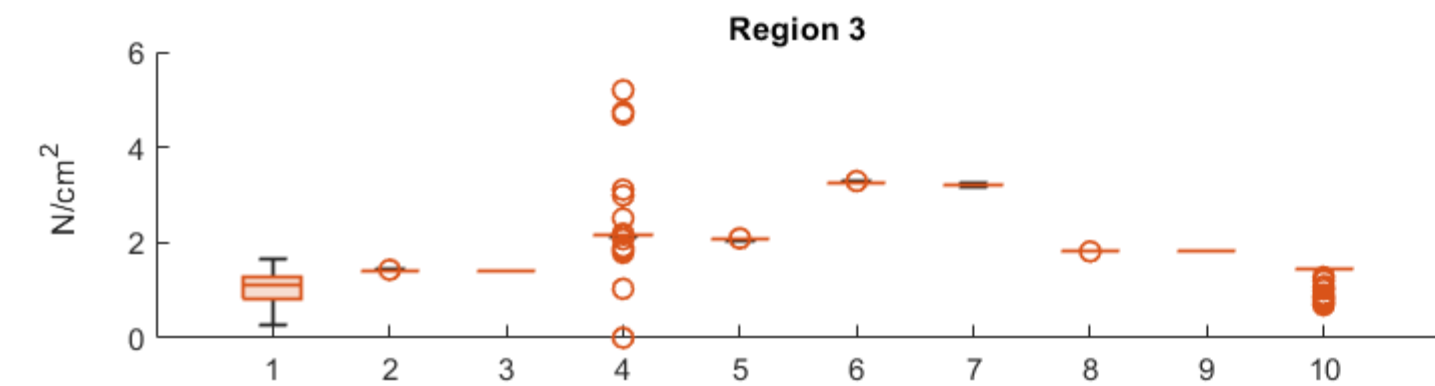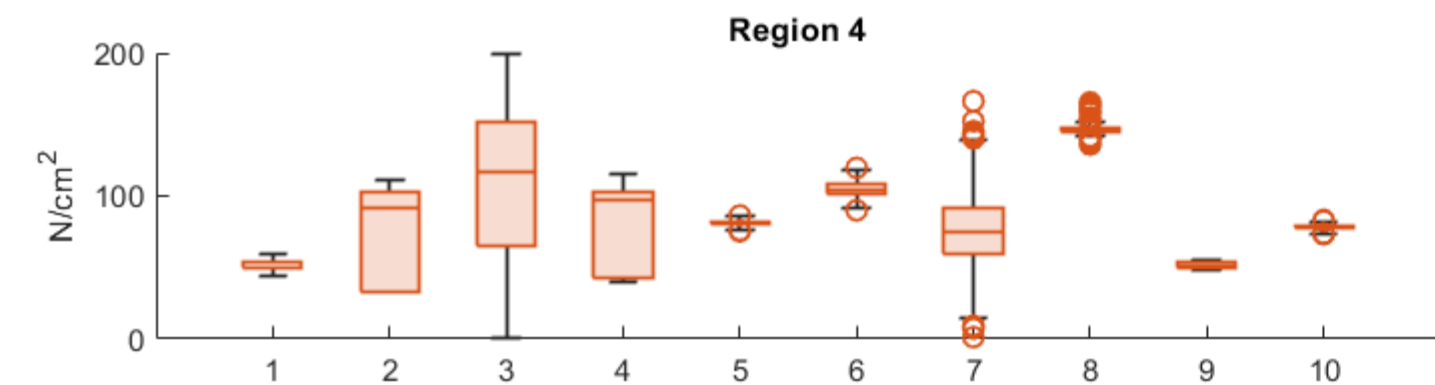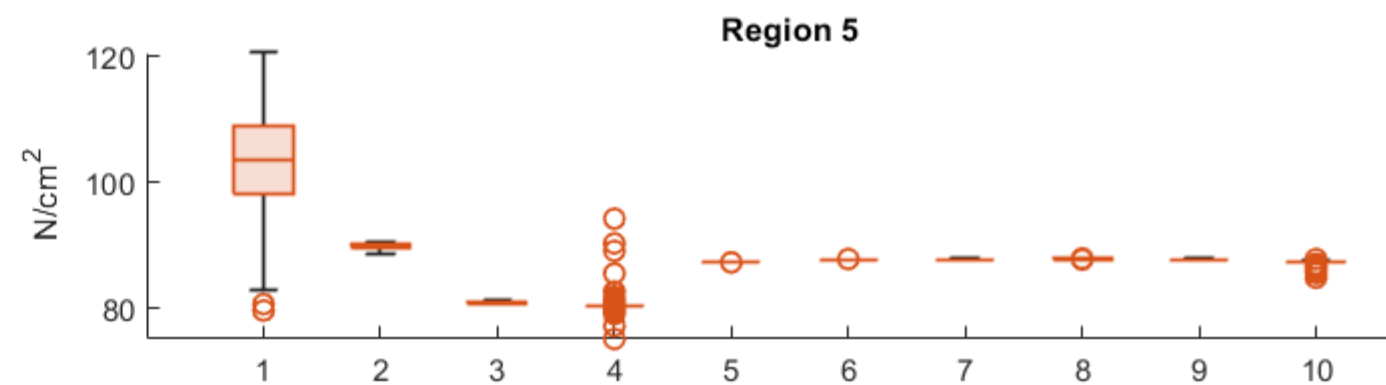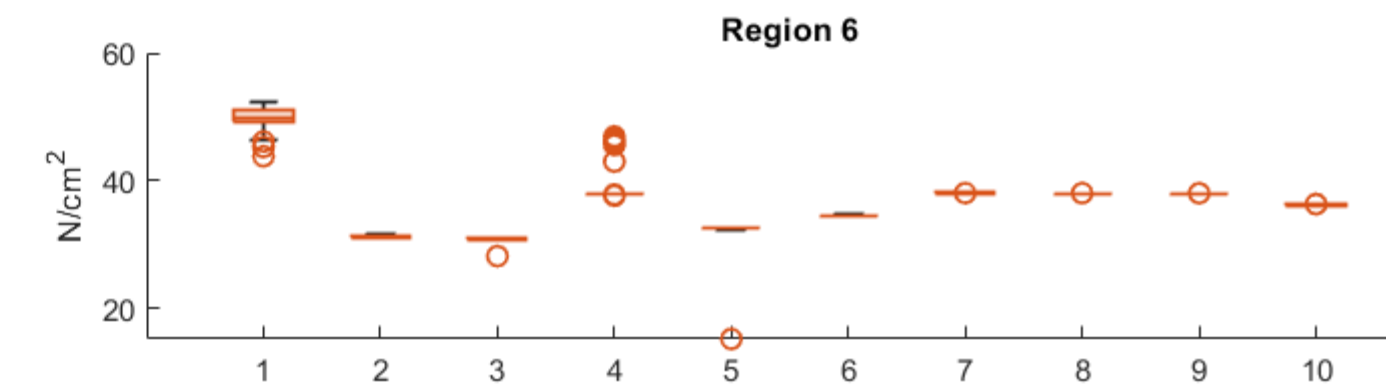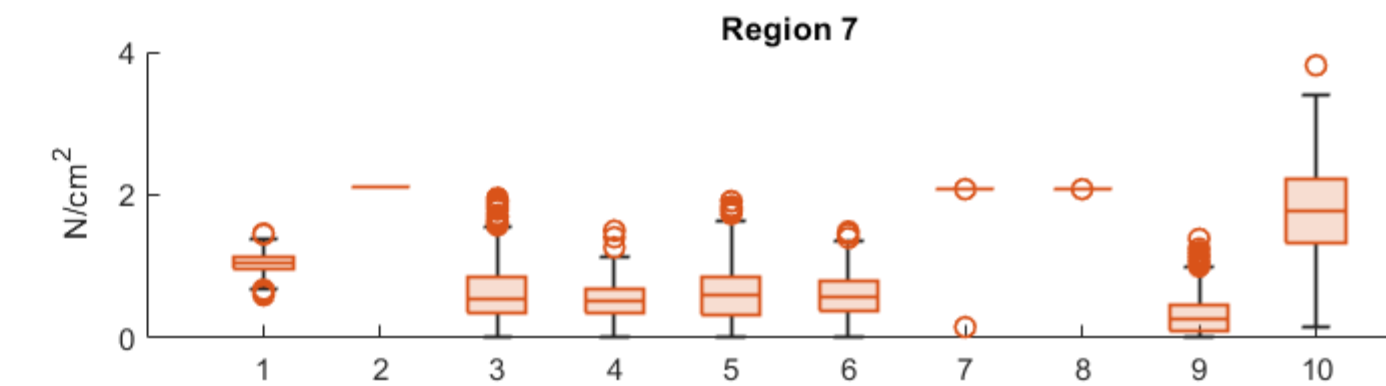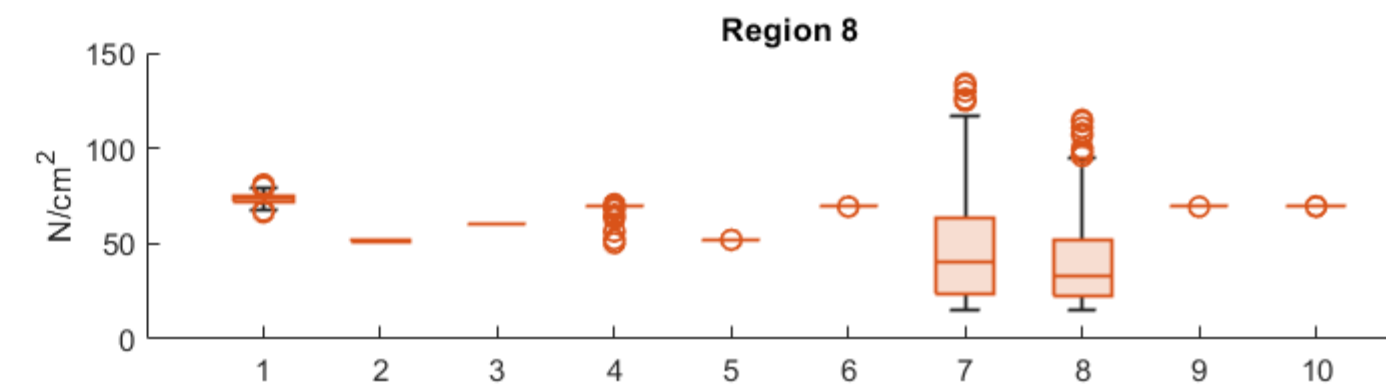

Supplement: S2 Fig — (PDF) [file pone.0280505.s002.pdf]

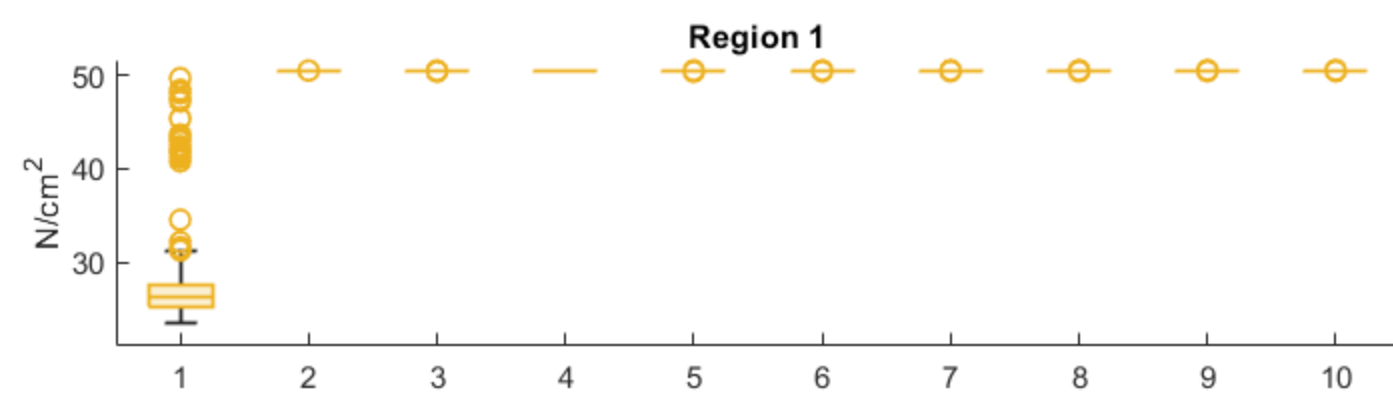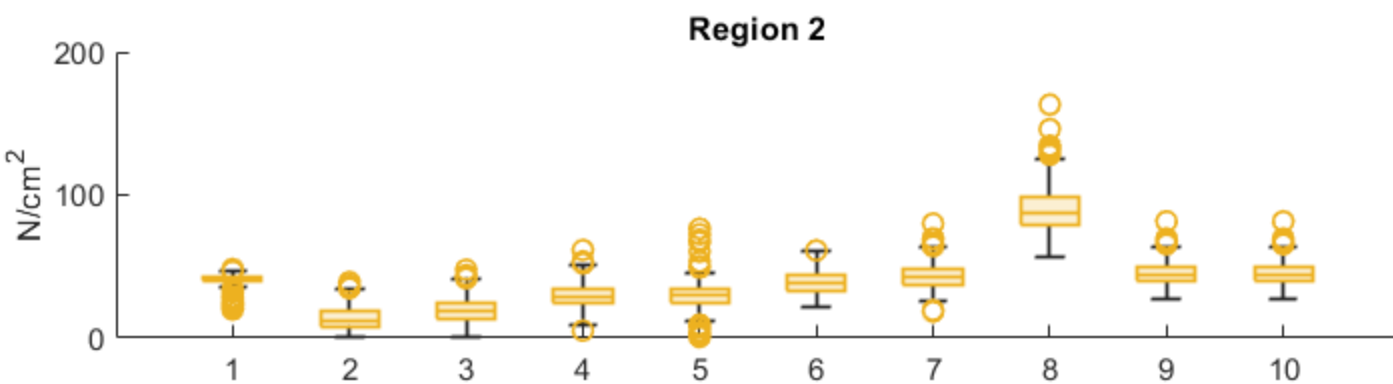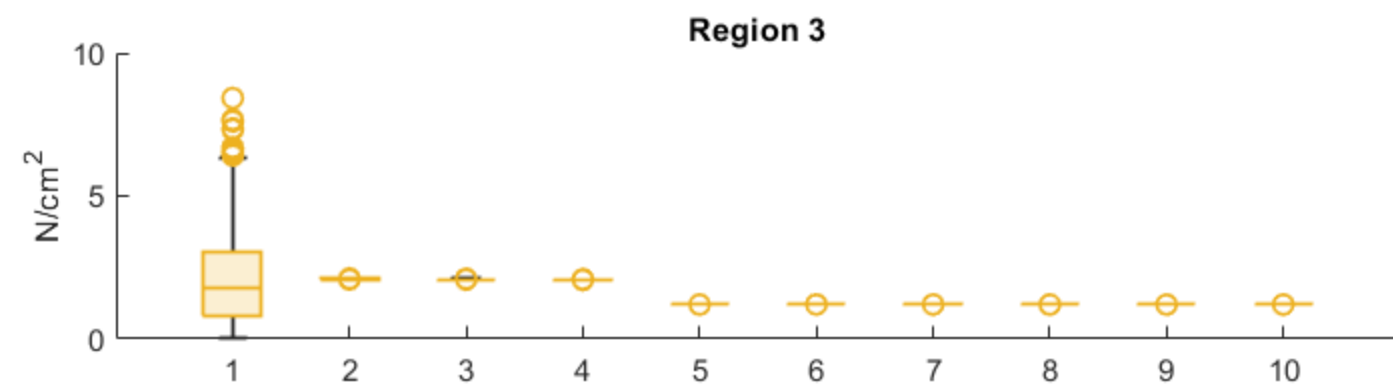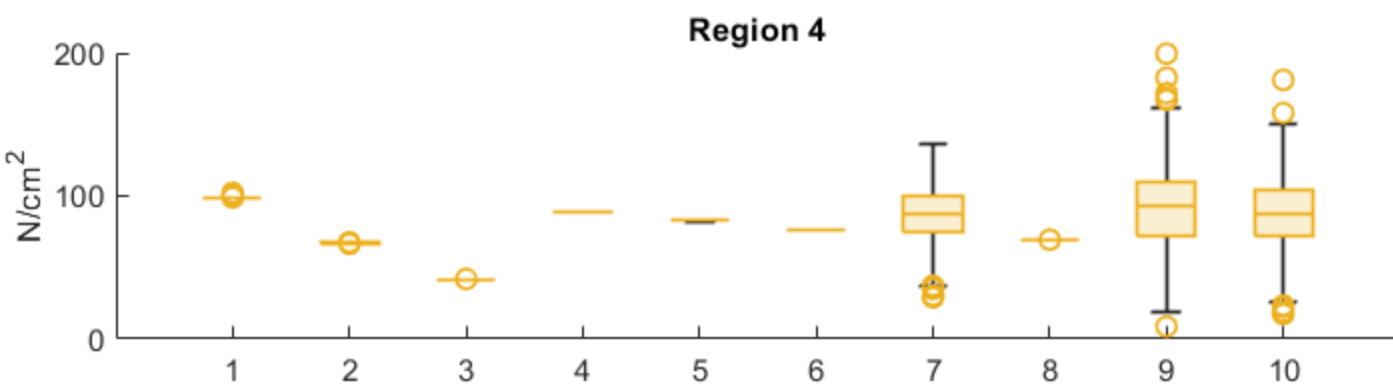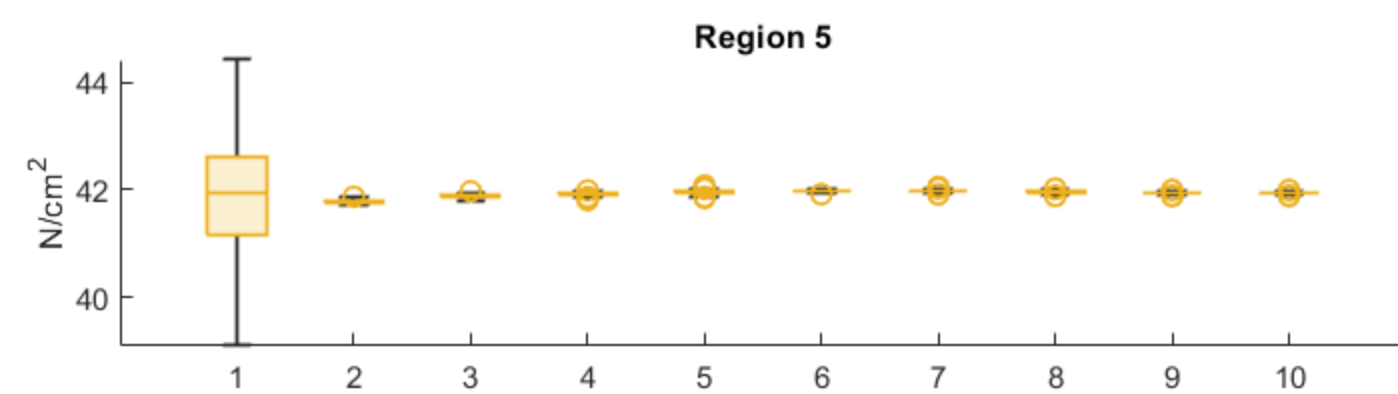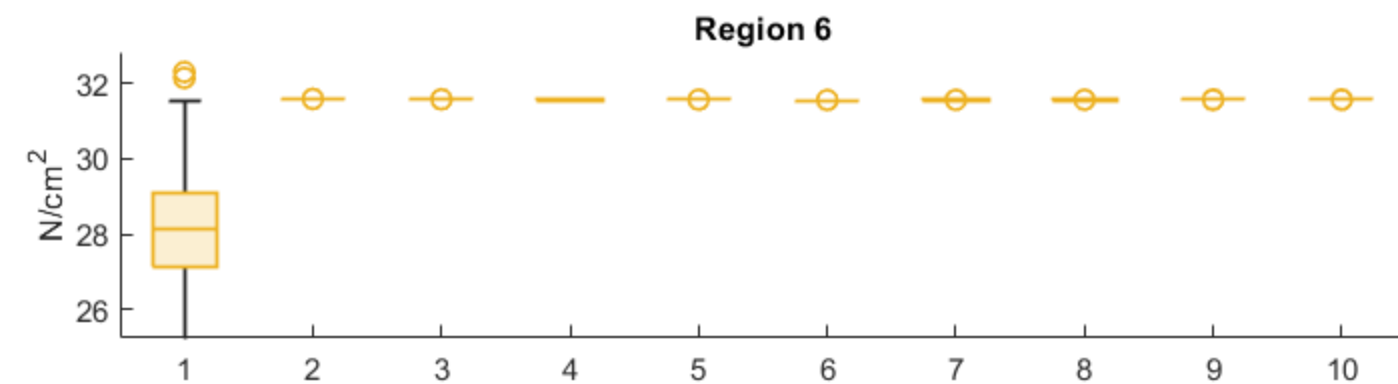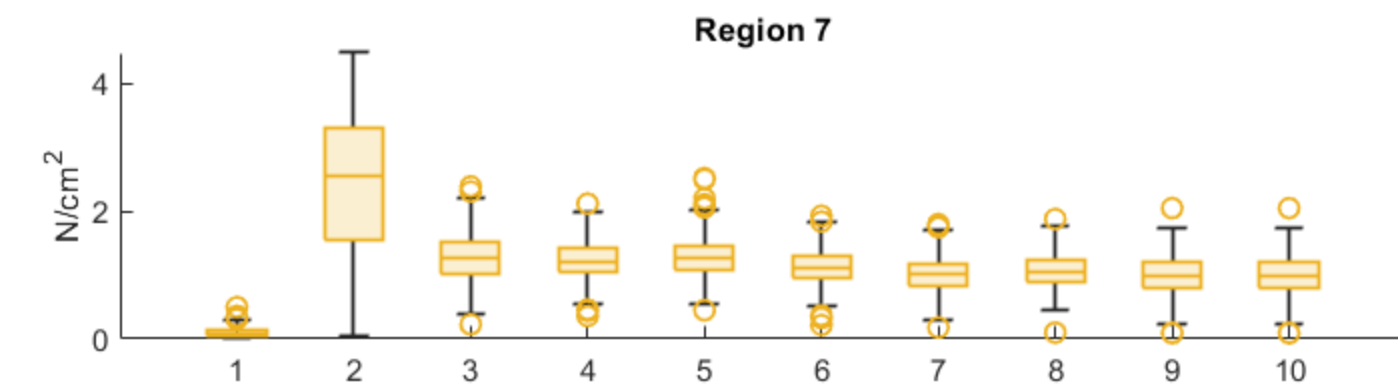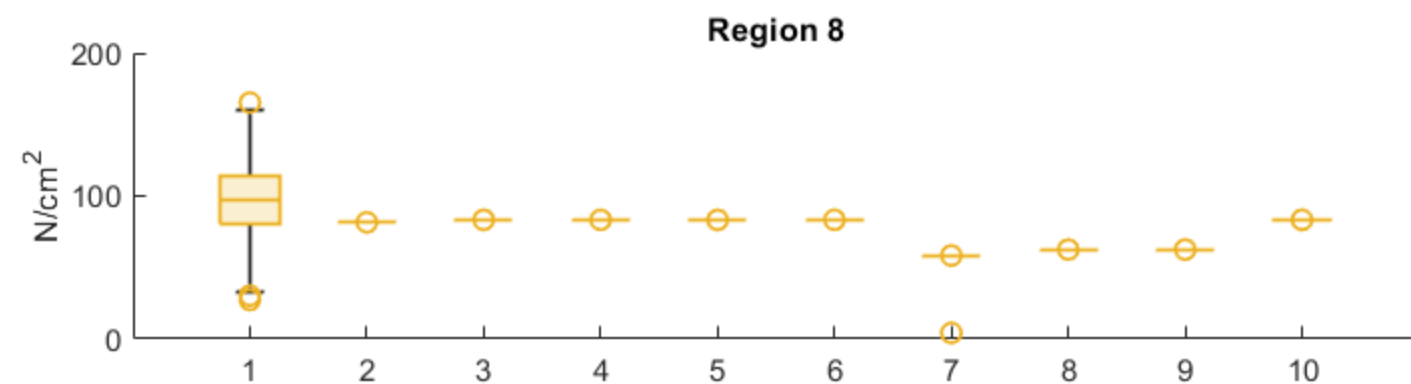

Supplement: S3 Fig — (PDF) [file pone.0280505.s003.pdf]

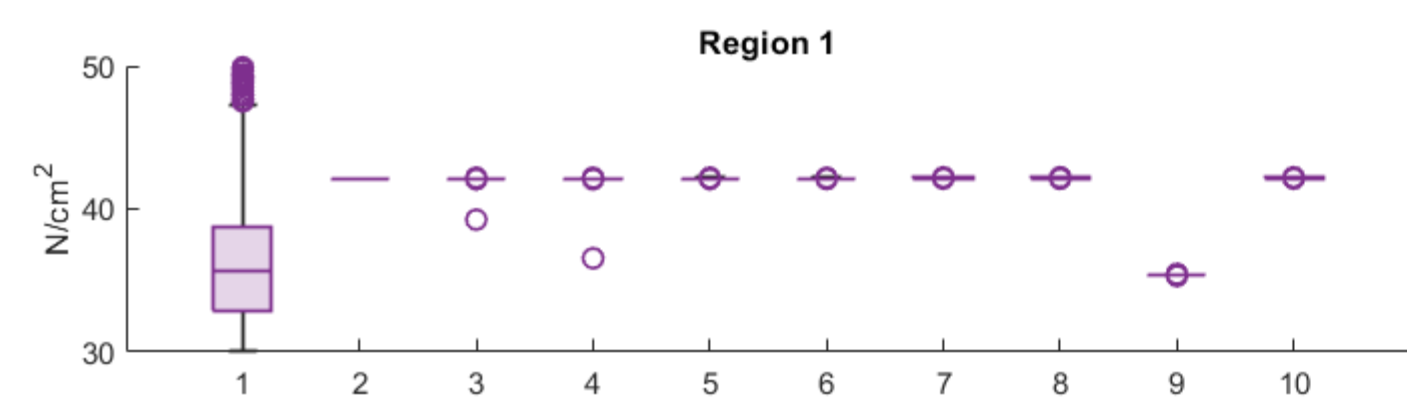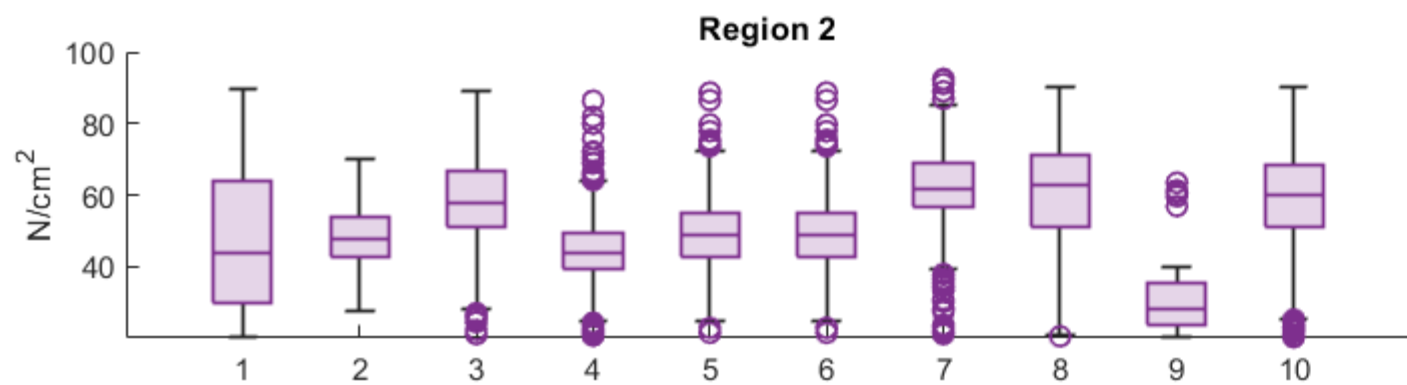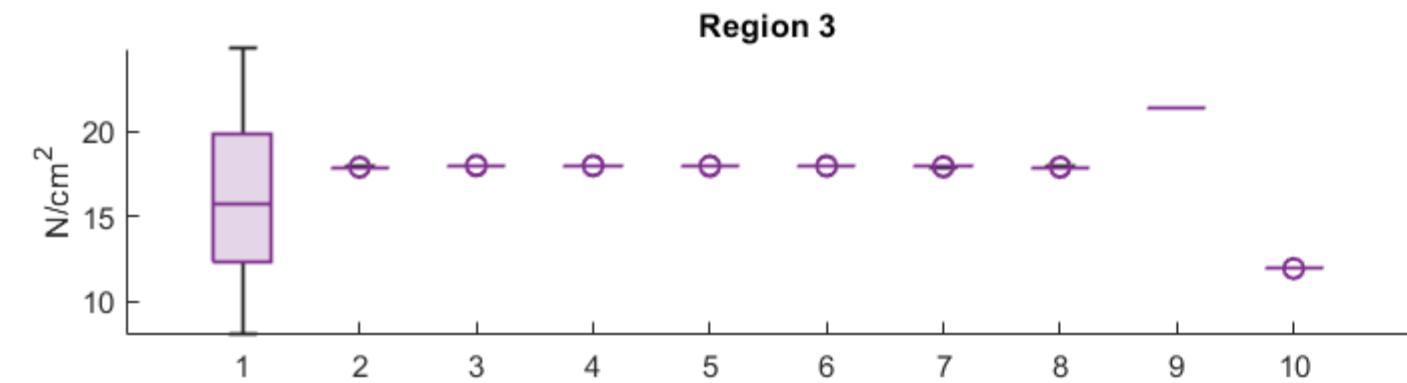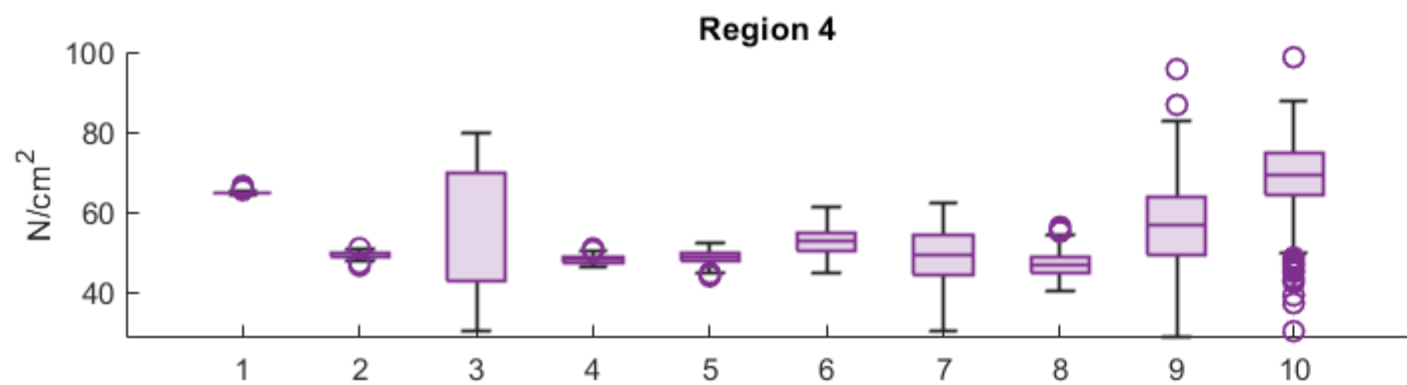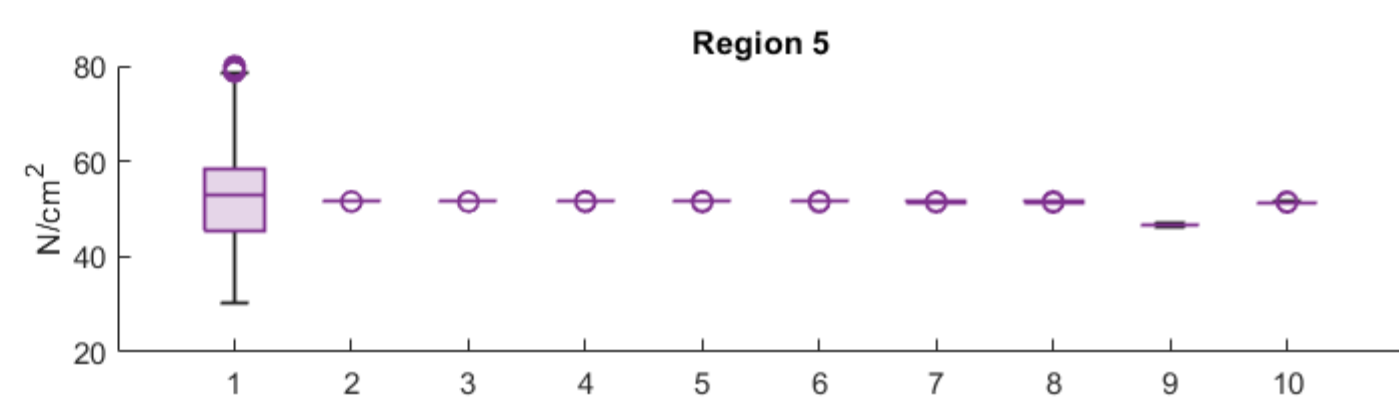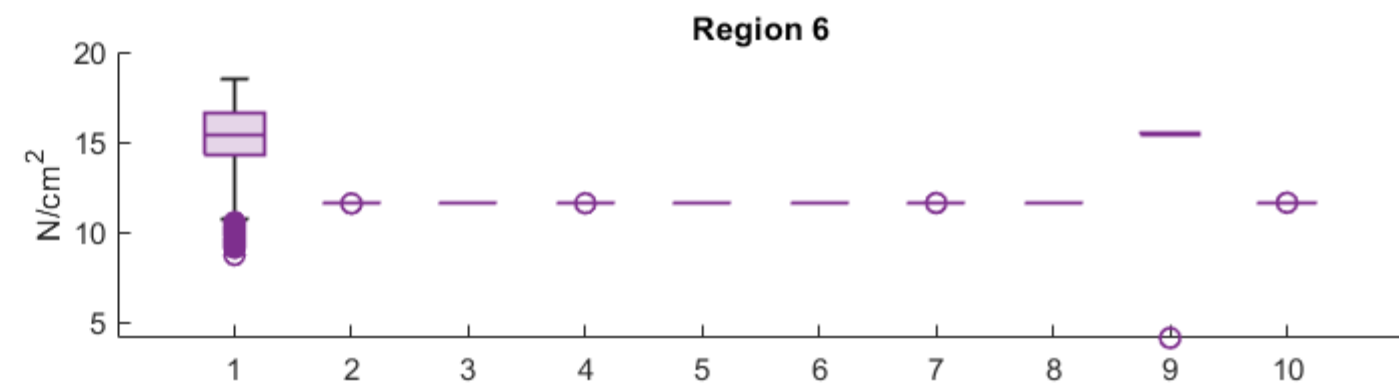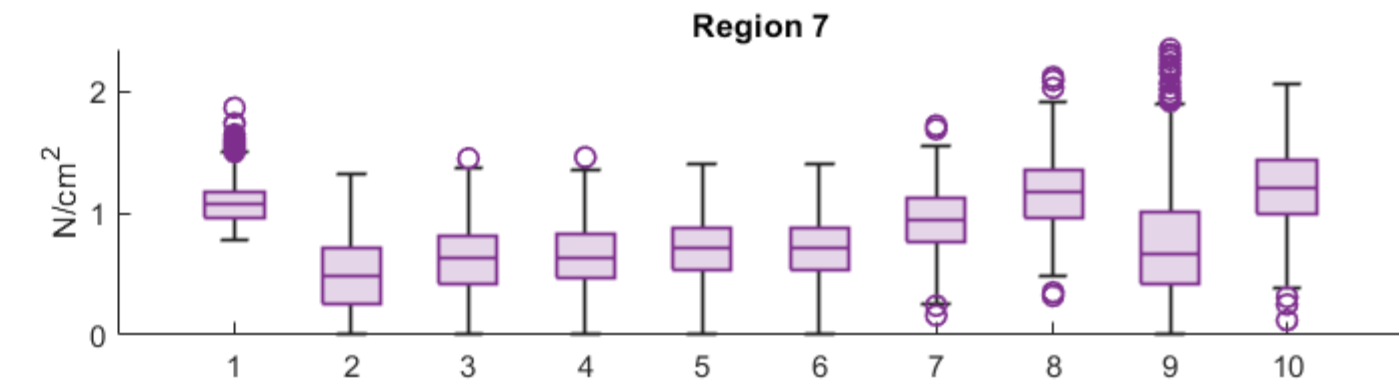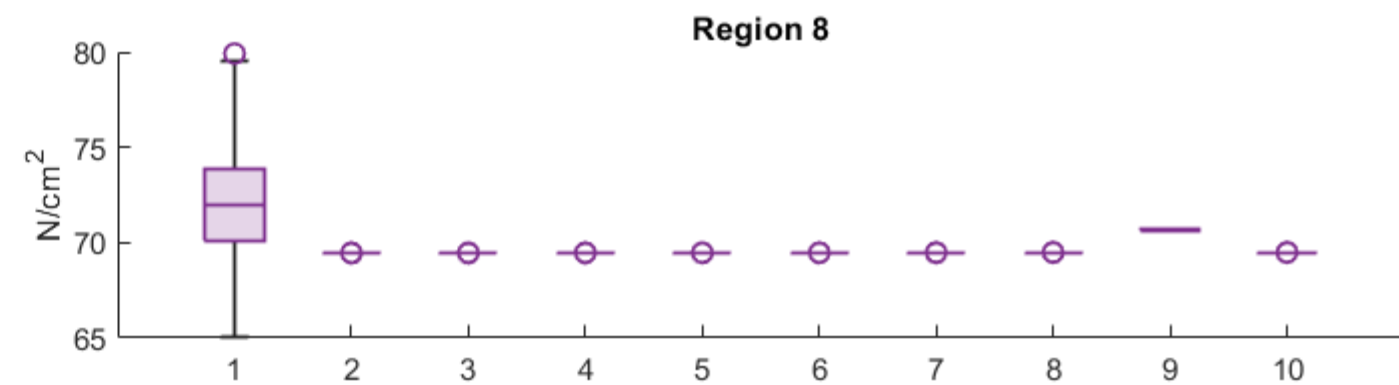

Supplement: S4 Fig — (PDF) [file pone.0280505.s004.pdf]

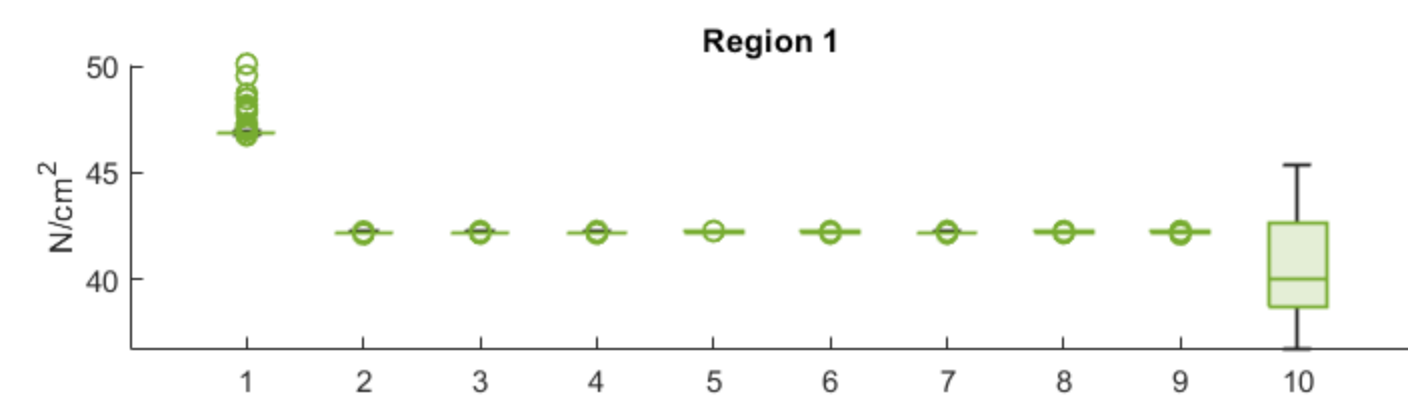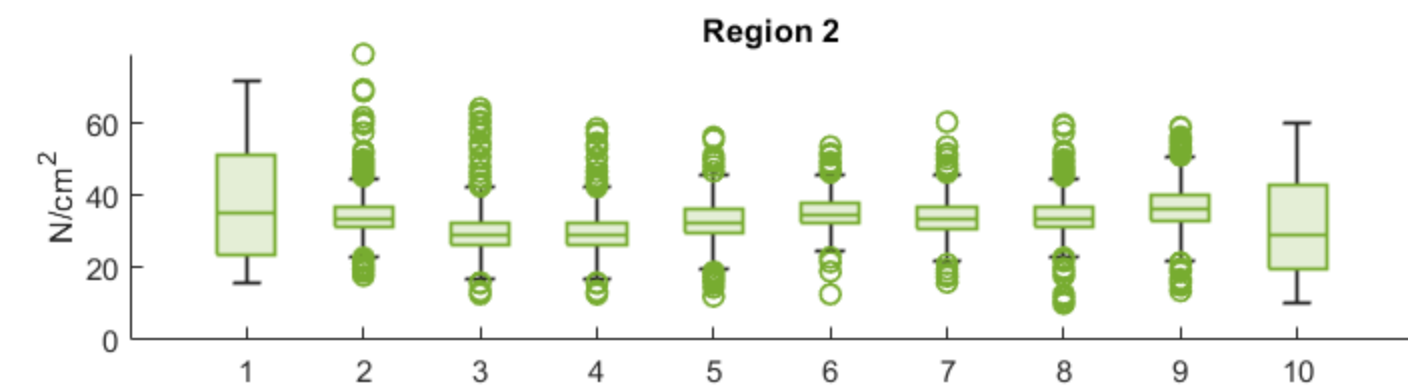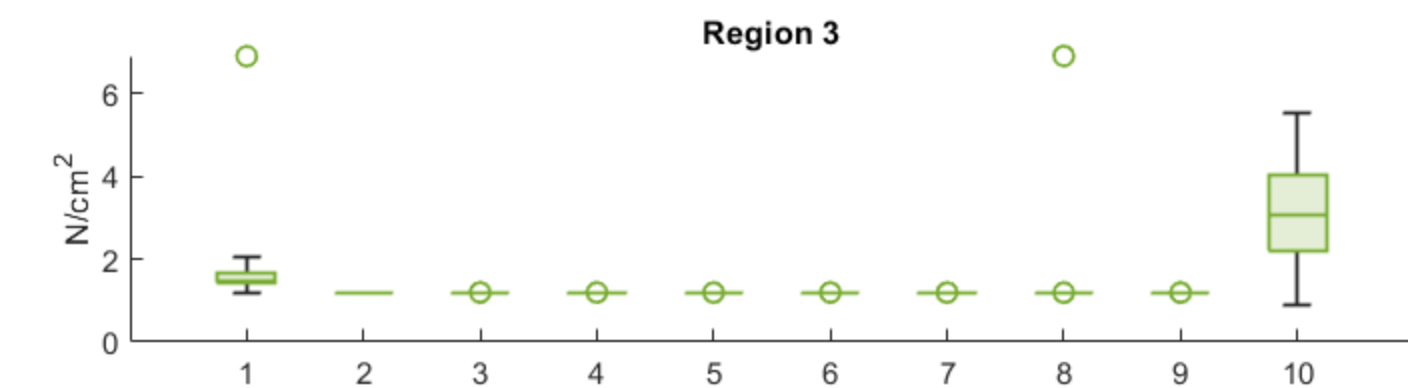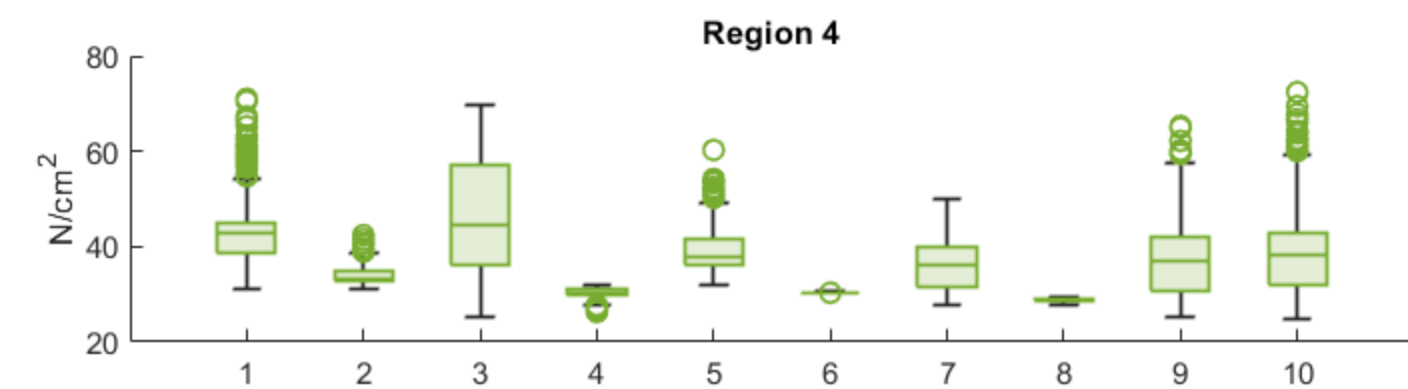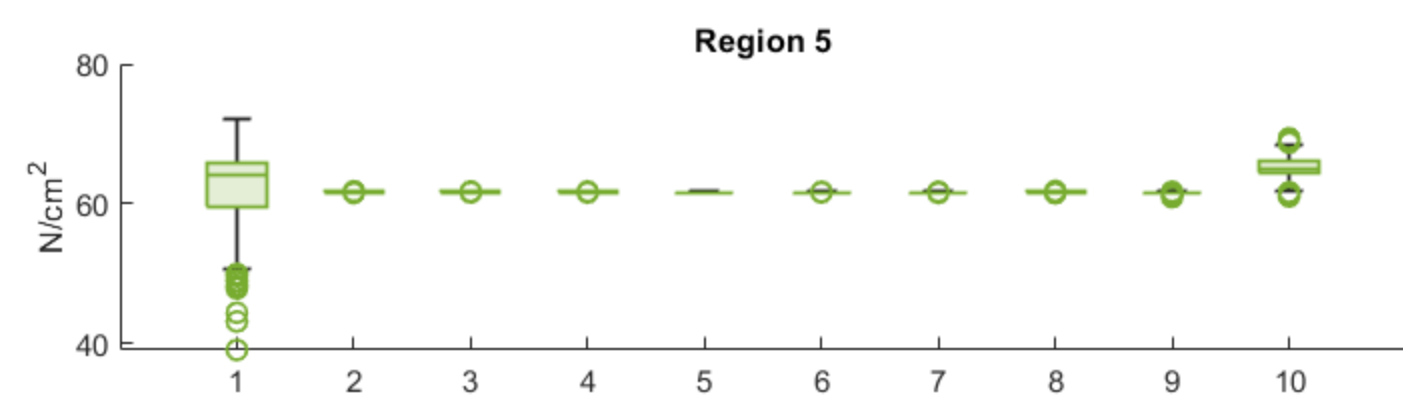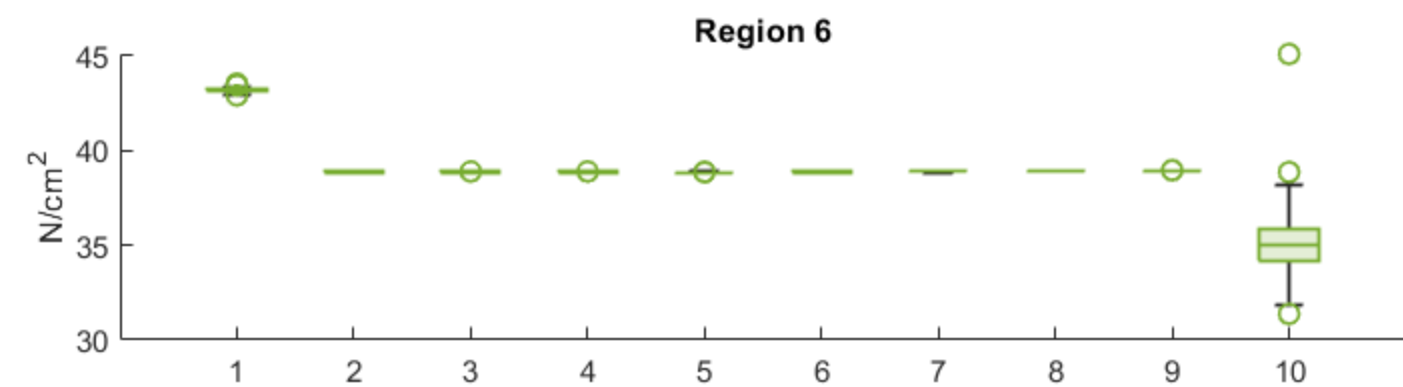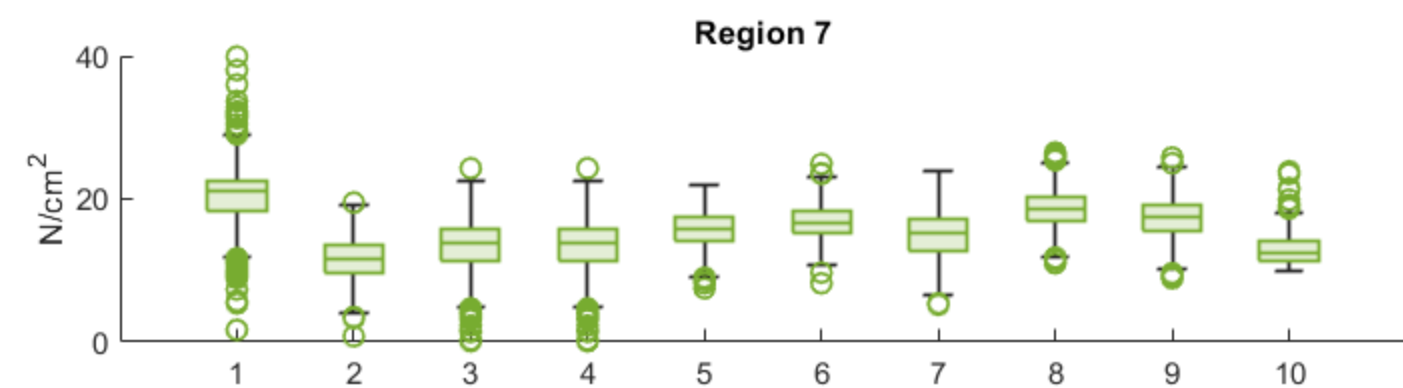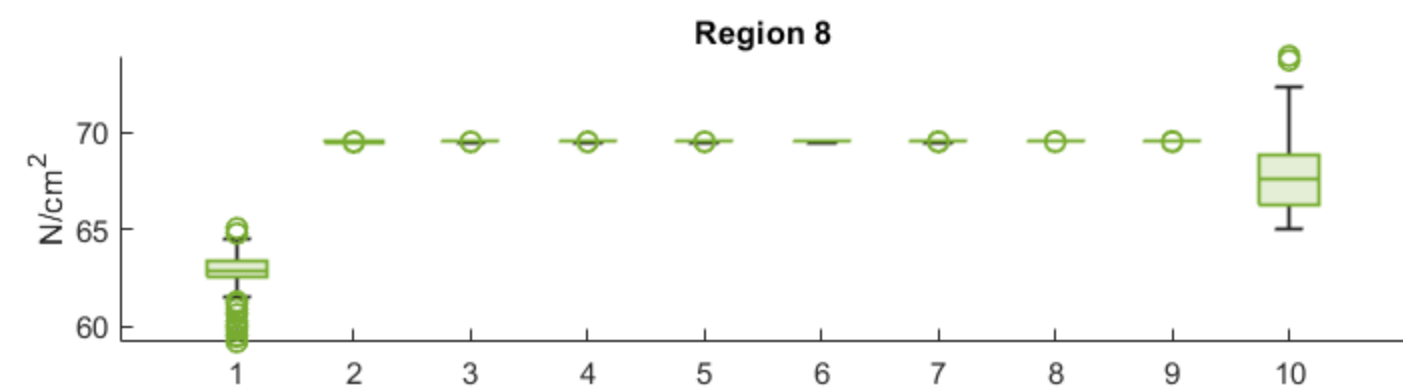

Supplement: S5 Fig — (PDF) [file pone.0280505.s005.pdf]
